# Serum and urinary monocyte chemoattractant protein-1 as markers of inflammation and renal damage in dogs with naturally occurring leishmaniosis

**DOI:** 10.1186/s13071-024-06432-0

**Published:** 2024-08-29

**Authors:** Valeria Pantaleo, Tommaso Furlanello, Laura Ventura, Laia Solano-Gallego

**Affiliations:** 1 San Marco Veterinary Clinic and Laboratory, Veggiano, Padua Italy; 2 Department of Statistical Sciences, University of Padova, Padua, Italy; 3 Departament de Medicina i Cirurgia Animals, Universitat Autònoma de Barcelona, Bellaterra, Barcelona, Spain

**Keywords:** Canine, *Leishmania infantum*, Renal disease, LeishVet, IRIS, Clinical staging

## Abstract

**Background:**

Renal disease in canine leishmaniosis is of great importance owing to increased risk of mortality. In human visceral leishmaniosis, monocyte chemoattractant protein-1 (MCP-1) has been used as a marker of renal damage and inflammation. The purpose of this study was first to determine the serum MCP-1 and urinary MCP-1-to-creatinine ratio (uMCP-1/Cr) in healthy dogs and dogs with leishmaniosis at diagnosis, and second to determine whether these markers can differentiate disease severity at diagnosis.

**Methods:**

In total, 19 healthy seronegative dogs and 38 dogs with leishmaniosis were included in the study. Dogs with leishmaniosis were classified as LeishVet clinical staging and as International Renal Interest Society (IRIS) staging. Serum and urinary MCP-1 concentrations were measured with an enzyme-linked immunosorbent assay. A receiver operating characteristic (ROC) curve determined disease severity at diagnosis between two LeishVet groups (Stage II versus stage III and IV).

**Results:**

Dogs in Leishvet stages IIb, III, and IV had a median serum MCP-1 and uMCP-1/Cr concentration higher than healthy dogs (*P* < 0.0001). No statistical differences were found in serum MCP-1 and uMCP-1/Cr between dogs in LeishVet stage IIa and healthy dogs. The dogs in LeishVet stage IV had significantly higher serum MCP-1 and uMCP-1/Cr compared with the dogs in LeishVet stage IIa (*P* < 0.0001). Serum MCP-1 and uMCP-1 were significantly higher in dogs in IRIS stage I and II + III + IV compared with healthy dogs. Dogs stage II + III + IV of IRIS had a significantly higher serum MCP-1 compared with dogs in IRIS stage I (*P* < 0.0001). The area under the ROC curve for serum MCP-1 was 0.78 [95% confidence interval (CI) 0.64–0.93] and for uMCP-1/Cr it was 0.86 (95% CI, 0.74–0.99). The optimal cutoff value for serum MCP-1 and uMCP-1/Cr was 336.85 pg/ml (sensitivity of 79% and specificity of 68%) and 6.89 × 10^−7^ (sensitivity of 84% and specificity of 79%), respectively.

**Conclusions:**

Serum MCP-1 and uMCP-1/Cr are increased in dogs with leishmaniosis compared with healthy dogs, suggesting the presence of inflammation and renal injury. Serum MCP-1 and uMCP-1/Cr were more elevated in the advanced stages of the disease compared with the moderate stages and, therefore, can be markers of the severity of the disease process.

**Graphical Abstract:**

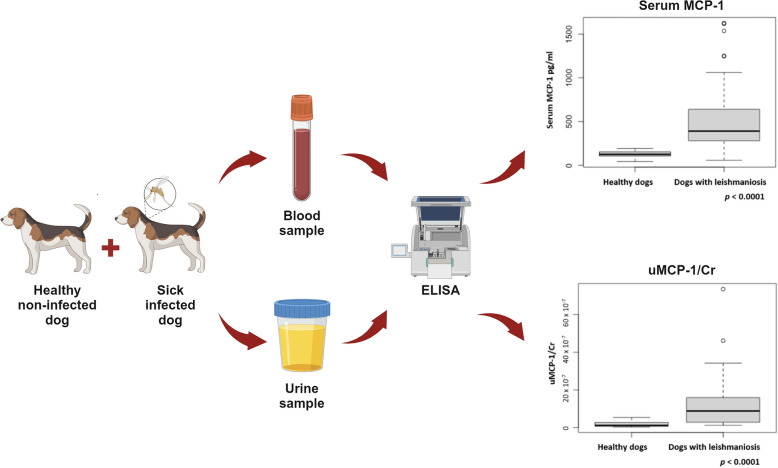

**Supplementary Information:**

The online version contains supplementary material available at 10.1186/s13071-024-06432-0.

## Background

*Leishmania infantum* is a protozoan parasite that can cause a wide spectrum of clinical manifestations in infected dogs, with skin lesions being the most frequent clinical manifestation among them and other general clinical signs depending on the organs involved [1]. Following transmission, parasites multiply in the skin at the infection site, can migrate into the viscera, and if they colonize the kidneys, or if the circulating immune complexes that formed are deposited in the kidneys, renal disease can develop and the eventual progression to chronic kidney disease may be fatal for the dog since chronic kidney disease is considered the main cause of death in dogs with leishmaniosis [2, 3].

*Leishmania infantum* infection in dogs can cause inflammation that varies according to the severity of the disease (based on the different clinical and clinicopathological findings) as shown by several studies on naturally occurring and experimental canine leishmaniosis (CanL) that demonstrated an increase in positive acute phase proteins, such as haptoglobin, C-reactive protein, ceruloplasmin, serum amyloid A, and ferritin, or a decrease in negative acute phase proteins, such as paraoxonase-1 and apoliprotein-A1, during various stages of the disease [4–7]. Acute phase proteins can change in concentration when systemic inflammation occurs [8] and in a variety of different infectious, inflammatory, and neoplastic diseases [9, 10]. Ideally, owing to the increased mortality risk of dogs with kidney disease in leishmaniosis, it would be desirable to have a biomarker capable of showing renal inflammation and damage before the development of established renal disease that can lead to a reduction in renal function over time.

Crucial for defense against *L*. *infantum* infection is the host’s ability to mount a cell-mediated immune response capable of controlling and/or eliminating the parasite [11]. During this process, various chemokines play a fundamental role in attracting specific leucocytes, participating in cell-mediated-immunity, cell activation, and antileishmanial activity [12, 13]. In human leishmaniasis, cytokine / chemokine concentrations are modulated differently depending on the clinical forms of the disease and the causative species of *Leishmania* [14]. In dogs, immune control of *L*. *infantum* requires a balance between proinflammatory T helper 1 type CD4 + cells to control parasite replication and T regulatory 1 cells that mediate an immunosuppressive regulatory response that leads to leishmaniosis progression [15].

Monocytes chemoattractant protein-1 (MCP-1) is a member of the C–C family of chemokines that mobilizes monocytes from the bone marrow to the site of inflammation. Many types of cells, such as monocytes, fibroblasts, astrocytes, mast cells, and endothelial cells, produce MCP-1 that responds to inflammation [16–19]. Monocytes chemoattractant protein-1 functions as a chemoattractant by binding to its receptor in monocytes and macrophages [20]. Several reports demonstrated the role of MCP-1 in various chronic inflammatory conditions, such as atherosclerosis, rheumatoid arthritis, glomerulonephritis, pulmonary hypertension, and pulmonary fibrosis, in human patients [21]. In dogs, MCP-1 has been evaluated in different biological substrates, such as blood [22–37], urine [38, 39], cerebrospinal fluid [40–42], synovial fluid [43–45], and bronchoalveolar lavage [28]. Most veterinary studies have shown the utility of measuring serum MCP-1 as an inflammatory marker in many inflammatory diseases, such as primary immune-mediated hemolytic anemia, pemphigus foliaceus, and suspected pancreatitis [23, 32, 35], neoplastic diseases, such as lymphoma, hemangiosarcoma, and urothelial carcinoma [24, 36, 39], and infectious diseases, such as babesiosis due to *Babesia canis* and *Babesia rossi*, and coccidiomycosis [30, 31, 34]. In human medicine, urinary MCP-1 (uMCP-1) has been associated with kidney damage and inflammation in acute and chronic renal diseases [46–50]. Previous data showed a correlation between elevated uMCP-1 and inflammation represented by macrophages in the renal tissue in human patients [51]. However, there is scarce information on the use of urinary MCP-1 in the detection of acute or chronic kidney damage in dogs [38, 39, 52].

Various human studies have evaluated the usefulness of MCP-1 in cutaneous and visceral leishmaniosis [14, 53–57]. A human study evaluated serum MCP-1 in patients with cutaneous leishmaniosis [58] and only one study evaluated uMCP-1 in patients with visceral leishmaniosis and showed an increase in uMCP-1 normalized to creatinine in those patients compared with healthy patients [59]. In CanL, some studies have evaluated MCP-1 as a marker of the immune response against *Leishmania*. Strauss-Ayali et al. evaluated the expression of MCP-1 and other chemokines in the spleen in naturally and experimentally *L. infantum* infected dogs [60]. Some authors showed higher expression of MCP-1 and other chemokines on the skin of dogs with visceral leishmaniosis [61]. Another study evaluated the expression of MCP-1 in the liver and spleen of dogs with visceral leishmaniosis [62]. In 2021, Verçosa and colleagues evaluated apoptosis and MCP-1 expression in renal tissues of *Leishmania*-infected dogs [63]. Interestingly, an elevation of MCP-1 messenger RNA in renal tissues has recently been studied in CanL, which is associated with infection in dogs from Brazil [64].

Unfortunately, to the best knowledge of the authors, the determination of serum MCP-1 and uMCP-1 in dogs with leishmaniosis has not yet been documented. For these reasons, the aims of the present study were: (1) to determine the serum MCP-1 and the uMCP-1-to-creatinine ratio (uMCP-1/Cr) in healthy dogs and in dogs with leishmaniosis in different clinical stages of the disease [according to the LeishVet and International Renal Interest Society (IRIS) stagings] [65–67] using a commercial enzyme-linked immunosorbent assay (ELISA); (2) to assess whether serum MCP-1 and uMCP-1/Cr can differentiate the severity of the disease (based on the LeishVet classification) at the time of diagnosis; and (3) to evaluate the correlation between serum MCP-1 and various inflammatory and renal biomarkers, and the correlation between uMCP-1/Cr and various renal and urinary biomarkers at diagnosis.

## Methods

### Dogs

This is a cross-sectional study that includes 57 client-owned dogs that were admitted to the San Marco Veterinary Clinic (Veggiano, Italy) for various medical reasons between May and October 2023.

Two study groups were defined as healthy dogs (*n* = 19) and dogs with leishmaniosis (*n* = 38). The following inclusion criteria were required to be considered healthy dogs: (1) unremarkable physical examination; (2) normal results in all laboratory tests, including complete blood count (CBC), serum biochemistry, coagulation profile, and urinalysis; (3) a negative *L*. *infantum* serology; (4) no history of recent illness; and (5) no drug administration at the time of evaluation. The diagnosis of clinical leishmaniosis was based on compatible clinical signs, clinicopathological findings, a positive *L*. *infantum* ELISA serology, and a positive *Leishmania* real-time polymerase chain reaction (q-PCR) in the bone marrow [2, 65]. All dogs were diagnosed for the first time with clinical leishmaniosis. The following inclusion criteria were required for dogs with leishmaniosis: (1) never treated with conventional anti-*Leishmania* drugs or immunomodulators, such as domperidone or nucleotides and AHCC or vaccine against leishmaniosis; (2) routine laboratory tests including CBC, serum biochemistry, coagulation profile, urinalysis, and abdominal ultrasound; (3) absence of *Dirofilaria immitis* antigen (Filarcheck 96, biopronix by Agrolabo, Italy), absence of *Anaplasma phagocytophilum*, *Ehrlichia canis*, and *Rickettsia conorii* antibodies (semiquantitative immunofluorescence by MegaFLUO ANAPLASMA ph. MEGACOR; MegaFLUO EHRLICHIA canis MEGACOR; MegaFLUO RICKETTSIA conorii MEGACOR; Hörbranz, Austria); (4) inactive urine sediment; (5) no other concurrent diseases; and (6) no medication administration in the previous 3 months with the exception of repellent or antiparasitic drugs.

During physical examination, after an adequate acclimatization period to the environment, in all dogs enrolled in the study, systolic blood pressure (SBP) was measured with the automated blood pressure monitor for companion animals SunTech Vet 20 (SunTech Medical Inc., USA), and the average value of four consecutive measurements was recorded. Laboratory blood and urine tests were carried out in the morning after 12 h of fasting without pharmacological or other restrictions.

Once the diagnosis of leishmaniosis was made, all dogs were classified according to LeishVet classification [2, 65, 66], and the recommendations of the International Renal Interest Society (IRIS) for chronic kidney disease [67]. Subsequently, dogs were divided into group 1 if they belong to LeishVet stage IIa or IIb (moderate disease), and group 2 if they belong to LeishVet stage III or IV (severe to very severe disease). According to IRIS staging, after classifying each dog in the stage to which it belongs, dogs were considered in IRIS stage I or aggregated in IRIS stages II + III + IV for statistical analysis.

### Blood tests

All tests were performed at the San Marco Veterinary Laboratory (Veggiano, Italy). A blood sample was collected by cephalic or saphenous or jugular venipuncture in a 10 ml sterile plastic syringe. Then, 2 ml of blood was transferred to plastic tubes containing K_3_-EDTA for a CBC performed on an automated hematology analyzer (ADVIA 2120i, Siemens, Germany) with a blood smear reading. A total of 4 ml of blood was placed in serum glass tubes for chemistry analysis performed in an automated biochemical analyzer (Atellica® Solution, Siemens, Germany), and 2 ml of blood was placed in plastic tubes containing 3.2% sodium citrate for coagulation profile performed in an automated coagulation analyzer (BCSXP, Siemens, Germany). The following parameters were evaluated: paraoxonase-1 (PON-1), haptoglobin (Hp), ferritin (Ft), C-reactive protein (CRP), total iron binding capacity (TIBC), iron, albumin (Alb), globulins (Glob), urea, and creatinine (Cr). In addition, symmetric-dimethylarginine (SDMA) was measured with a canine SDMA ELISA (Eurolyser Diagnostica GmbH, Salzburg, Austria).

To detect *L*. *infantum* antibodies, a *Leishmania* ELISA test was performed following the manufacturer’s instructions (VetLine *Leishmania*, *Leishmania* ELISA test, NovaTec Immunodiagnostica GmbH, Dietzenbach, Germany) [68]. The result of the *Leishmania* ELISA test was considered negative if the antibody level was < 9%, doubtful if the antibody level was 9–11%, and positive if the antibody level was > 11%.

### Urine examination

Urine was collected at the time of the visit (in the morning after blood sampling) by free catch in a sterile container in all dogs. A total volume of 10 ml of urine was obtained during spontaneous urination, and 7 ml of urine was used for urinalysis and urinary chemistry performed on an automated urine analyzer (CLINITEK Novus^®^, Siemens, Germany) and on an automated biochemical analyzer (Atellica^®^ Solution, Siemens, Germany), respectively. Whole urine was used for urine analysis with test strips (CLINITEK Novus Pro12 Urinalysis Cassette, Siemens, Germany), urine specific gravity measurement (USG) (CLINITEK Novus Pro12 Urinalysis Cassette, Siemens, Germany), urine protein to creatinine ratio (UPC) determination (calculated by dividing the concentration of urinary proteins by the concentration of urinary Cr concentration), and urinary chemistry. Urine proteins (UPs) were measured in an automated spectrophotometer (Atellica^®^ Solution, Siemens, Germany) using pyrogallol red (Atellica CH Urinary/Cerebrospinal Fluid Protein (UCFP), Siemens Healthcare Diagnostics Inc., USA), and uCr with a modified Jaffe method (Siemens Healthcare Diagnostics Inc., USA). Samples were automatically prediluted 1:5 to fit the linearity of the method according to manufacturer’s instructions. Urinary sediment was examined by a clinical pathologist with an optical microscope and only dogs with an inactive urine sediment [< 5 white blood cells per high power field (hpf), < 5 red blood cells/hpf or no visible bacteria] were considered for urinary podocin and nephrin measurements. The following parameters in the urine were evaluated: USG, UPC, urinary amylase to creatinine ratio (uAm/Cr), and urinary creatinine (uCr) concentration.

### MCP-1 determination

After centrifugation of blood and urine, serum and urinary supernatant were aliquoted in plastic cryotubes, frozen within 2 h after collection and stored at −80 °C until a canine CCL2/MCP-1 Quantikine ELISA Kit (R&D System, Minneapolis, MN, USA) was performed [69]. Once all samples were collected, the ELISA tests to detect serum and urinary MCP-1 were performed according to the manufacturer’s instructions. Briefly, an ELISA plate was set for control, standards, and sample wells. At first, 50 μL of the RD1W assay diluent was added to each well; subsequently, 50 μL of standard, control, or sample per well was added and left to incubate for 2 h at room temperature. After incubation, each well was aspirated and washed with 400 μL of wash buffer for a total of five washes. Then, 100 μL of canine MCP-1 conjugate was added to each well, incubated for 2 h at room temperature, and, again, after incubation, each well was aspirated and washed a total of five times. At this point, 100 μL of substrate solution was added to each well and incubated (protected from light) for 30 min at room temperature. At the end of the incubation, 100 µL of stop solution was added to each well to terminate the reaction. The optical density was determined with a microplate reader at a wavelength of 450 nm in 30 min. The standard curve for MCP-1 started from 1000 pg/mL and two-fold dilutions were made until 15.6 to pg/ml was obtained. Each sample was measured in duplicate, and the average values obtained were expressed in pg/ml. The standard curve was calculated using a computer-generated spline logistic curve fit with programme test result by the automatic analyzer Stratego (Futurlab ®, Limena, Padova, Italy). Once the concentration of uMCP-1 was determined, its levels were assessed relative to the concentration of uCr as uMCP-1/Cr to correct for dilution as reported in previous studies [70, 71].

### Evaluation of *Leishmania* parasitic load

*Leishmania* q-PCR was measured in the bone marrow of all dogs with leishmaniosis. Bone marrow aspirates were obtained from costochondral junctions using an 18-gauge needle connected to a 10 ml syringe according to the protocol described by Paparcone et al. for the diagnosis of CanL [72] DNA extraction was performed using a High Pure PCR Template Preparation Kit (Roche Science Applied) and carried out according to the manufacturer’s protocol. Real-time PCR was performed using LightCycler FastStart DNA Master^PLUS^ hybridization probes (Roche, Mannheim, Germany), using a LightCycler version 3.5.17 instrument (Roche, Mannheim, Germany). Commercial *L*. *infantum* primers and LC set hybridization probes (TIB Molbiol, Genova, Italy) that amplified a fragment of the kinetoplast minicircle were used. The thermal cycling was performed according to the manufacturer’s instructions (TIB Molbiol). Positive and negative controls were used in all q-PCR runs as previously reported [73].To be considered positive > 100 copies of kinetoplast/ml should be detected in bone marrow.

### Statistical analysis

Qualitative data were summarized using percentages. Quantitative variables were reported as mean and standard deviation (SD) or as median and interquartile range (IQR), according to the Shapiro–Wilk’s test for normality. Statistical differences between two groups were analyzed with Student’s *t*-test, under the hypotheses of normality and homoscedasticity, or with the Mann–Whitney rank test for nonnormal variables. For more than two groups, parametric or nonparametric analysis of variance (ANOVA) was applied, and post hoc analyses were performed through pairwise comparisons between group levels with Holm correction for multiple tests. The relationships between quantitative variables were evaluated using correlation plots. Finally, the receiver-operating characteristic (ROC) curve and the area under the ROC curve (AUC) were used to assess the ability of quantitative parameters to differentiate between the dogs in group 1 and the dogs in group 2 at the time of diagnosis. The accuracy of the test was classified as excellent (AUC > 0.9), good (0.8 < AUC ≤ 0.9), fair (0.7 < AUC ≤ 0.8), poor (0.6 < AUC ≤ 0.7), or failed (0.5 < AUC ≤ 0.6) [74]. The Youden procedure was applied to determine the best threshold value. The significance level was set at *P* < 0.05. Data were analyzed using the statistical software R version 4.3.2.

## Results

The results of demographic and clinical data, and the results of blood and urine analysis of all dogs are reported in Tables 1, 2, and 3. All data supporting the main conclusions are displayed in Additional File 1 (Dataset S1: signalment, clinical data, and serum and urinary parameters including MCP-1).

**Table 1:** Demographic and clinical data of healthy dogs and dogs with leishmaniosis

Parameter (units)	Healthy dogs	Dogs with leishmaniosis	Statistical analysis
	*n* = 19	*n* = 38	
Female/male	7/12	18/20	$\chi^{2}$= 0.22, *df* = 1, *P* = 0.64
Breed/ mixed breed	6/13	15/23	$\chi^{2}$ = 0.72, *df* = 1, *P* = 0.72
Castrated/intact	9/10	20/18	$\chi^{2}$ = 0.92, *df* = 1, P = 0.92
Age (months)	70.9 ± 40.0	71.4 ± 29.2	*t* = 0.22, *df* = 1, *P* = 0.96
Body weight (kg)	25.7 ± 12.9	22.2 ± 10.7	*t* = 1.11, *df* = 55, *P* = 0.27
Heart rate (bpm)	106.8 ± 20.8	119.1 ± 29.6	*t* = 1.61, *df* = 55, *P* = 0.11
Respiration rate (rpm)	24 (9)	32 (12)	*U* = 237.7, *P* = 0.04*
Systolic blood pressure (mmHg)	144 (25)	146.5 (28.5)	*U* = 259.5, *P* = 0.09

Data are expressed as numbers, mean ± standard deviation, or median and interquartile range

Kg, kilograms; bpm, beats per min; rpm, breaths per min; mmHg, millimeters of mercury; $\chi^{2}$, Chi-square test; *df*, degrees of freedom; *t*, t-test; *U*, Mann–Whitney *U*-test

^*^Statistically significant differences between healthy dogs and dogs with leishmaniosis

**Table 2:** Serum biochemistry results of healthy dogs and dogs with leishmaniosis

Parameter (units)	Healthy dogs	Dogs with leishmaniosis	Statistical analysis
(Reference interval)	*n* = 19	*n* = 38	
CRP (mg/dL)			
(0.01–0.07)	0.01 (0.00)	1.86 (4.64)	*U* = 57.5, *P* < 0.0001*
PON-1 (IU/L)			
(3.02–4.71)	3.59 (1.5)	3.25 (0.99)	*U* = 415, *P* 0.36
Ft (ng/mL)			
(80–272)	225 (68.5)	733 (715.8)	*U* = 65.5, *P* < 0.0001*
Hp (mg/dL)			
(2–165)	21 (60)	185 (204.3)	*U* = 92, *P* < 0.0001*
Iron (μg/dL)			
(95–213)	145 (34.5)	82 (55)	*U* = 569.5, *P* < 0.0001*
TIBC (μg/dL)			
(336–424)	362.1 (38.9)	294 (77.4)	*U* = 561.5, *P* < 0.0001*
Alb (g/dL)			
(2.9– 3.5)	3.3 (0.3)	2.4 (0.8)	*U* = 634.5, *P* < 0.0001*
Glob (g/dL)			
(2.9– 3.4)	3.2 (0.4)	4.7 (2.73)	*U* = 72, *P* < 0.0001*
Urea (mg/dL)			
(20–48)	33 (10.5)	34 (21.5)	*U* = 365.5, *P* = 0.95
Cr (mg/dL)			
(0.7–1.4)	1.17 (0.23)	0.87 (0.47)	*U* = 515, *P* = 0.009*
SDMA (μg/dL)			
(0–15)	9.87 (3.6)	15.81 (5.6)	*U* = 133.5, *P* < 0.0001*
Serum MCP-1 (pg/mL)	124.8 (48.1)	380.6 (345.4)	*U* = 62, *P* < 0.0001*

Data are expressed as mean ± standard deviation or median and interquartile range

CRP, C-reactive protein; PON-1, paraoxonase-1; Ft, ferritin; Hp, haptoglobin; TIBC, total iron binding capacity; Alb, albumin; Glob, globulins; Cr, creatinine; SDMA, symmetric-dimethylarginine; MCP-1, monocyte chemoattractant protein-1; *U*, Mann–Whitney *U*-test

^*^Statistically significant differences between healthy dogs and dogs with leishmaniosis

**Table 3:** Urine analysis results of healthy dogs and dogs with leishmaniosis

Parameter (Units)	Healthy dogs	Dogs with leishmaniosis	Statistical analysis
(Reference interval)	*n* = 19	*n* = 38	
USG			
(1015–1050)	1047.4 ± 10.8	1033.8 ± 13.4	*t* = 3.85, *df* = 55,* P* =0.0003*
UPC (mg/dl)			
(0.1–0.5)	0.2 (0.10)	0.75 (4.25)	*U* = 57.5, *P* < 0.0001*
uAm/Cr			
(0.1–50)	0.4 (0.45)	116.1 (1048.9)	*U* = 57, *P* < 0.0001*
uCr (mg/dL)			
(125–324)	302.5 (99.6)	154.4 (75.6)	*t* = 6.26, *df* = 55, *P* < 0.0001*
uMCP-1 (pg/mL)	357.4 (535.2)	1364.6 (1209.2)	*U* = 149, *P* = 0.0002*
uMCP-1/Cr × 10^–7^	1.21 (2.1)	8.85 (1.26)	*U* = 90, *P* < 0.0001*

Data are expressed as mean ± standard deviation or median and interquartile range

USG, urine specific gravity; UPC, urinary protein to creatine ratio; uAm/Cr, urinary amylase to creatinine ratio; uCr, urinary creatinine; uMCP-1, urinary monocyte chemoattractant protein-1; uMCP-1/Cr, urinary monocyte chemoattractant protein-1 to creatinine ratio; *t*, *t*-test; *df*, degrees of freedom; *U*, Mann–Whitney *U*-test

^*^Statistically significant differences between healthy dogs and dogs with leishmaniosis

A significant increase in respiration rate was found in dogs with leishmaniosis compared with healthy dogs even if the majority of leishmaniotic dogs had a respiration rate in the reference interval (Mann–Witney *U*-test, *U* = 237.5, *P* = 0.04, Table 1).

A significant increase in CRP, Ft, Hp, globulin, and SDMA and a decrease in albumin, iron, and TIBC was observed in leishmaniosis dogs compared with healthy dogs (*U* = 57.5, *P* < 0.0001;* U* = 65.5, *P* < 0.0001; *U* = 92, *P* < 0.0001; *U* = 72, *P* < 0.0001;* U* = 133.5, *P* < 0.0001; *U* = 634.5, *P* < 0.0001; *U* = 569.5, *P* < 0.0001; *U* = 561.5, *P* < 0.0001, respectively, Table 2). Serum MCP-1 was significantly higher in dogs with leishmaniosis compared with healthy dogs (*U* = 62, *P* < 0.0001, Table 2).

The UPC, uAm/Cr, uMCP-1, and the uMCP-1/Cr increased significantly in dogs with leishmaniosis compared with healthy dogs (*U* = 57.5, *P* = 0.0003; *U* = 57, *P* < 0.0001; *U* = 149, *P* = 0.0002; *U* = 90, *P* < 0.0001, respectively, Table 3).

According to the LeishVet guidelines, dogs with leishmaniosis were classified as stage IIa (*n* = 12), stage IIb (*n* = 7), stage III (*n* = 10), and stage IV (*n* = 9). Subsequently, all dogs were divided into group 1 (stages IIa and IIb, *n* = 19) and group 2 (stage III and IV, *n* = 19).

According to the IRIS staging of chronic kidney disease, dogs were classified as stage I (*n* = 31), stage II (*n* = 4), stage III (*n* = 1), and stage IV (*n* = 2), and subsequently aggregated as stage I (*n* = 31) and as stages II + III + IV (*n* = 7).

**Fig. 1:**
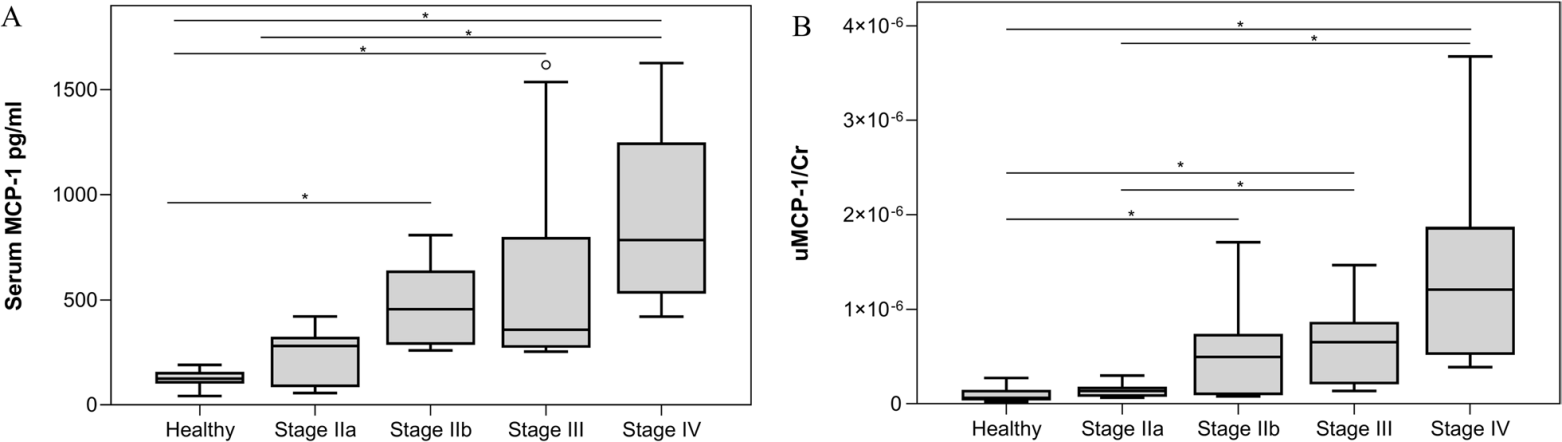
Box plots showing **A** serum MCP-1 and **B** uMCP-1/Cr in healthy dogs and dogs with leishmaniosis according to the LeishVet clinical staging system to which they belong. Stage IIa, LeishVet stage IIa; Stage IIb, LeishVet stage IIb; Stage III, LeishVet stage III; and Stage IV, LeishVet stage IV. *Statistically significant difference between the median of each group

Higher serum MCP-1 was observed in dogs in Leishvet stage IIb [median values and interquartile range (IQR): 455.9 pg/ml (290.17), Fig. 1A], III (median values and IQR: 357.6 pg/ml (246.76), Fig. 1A), and IV (median values and IQR: 784.9 pg/ml (664.24); Fig. 1A) compared with healthy dogs (median values and IQR: 124.83 pg/ml (48.05); Kruskall–Wallis H-test, *H* = 38.57, *df* = 4, *P* < 0.0001, respectively, Fig. 1A). Serum MCP-1 was also significantly higher in dogs in stage IV of Leishvet than in dogs in stage IIa (median values and IQR: 784.9 pg/ml (664.24) versus 280.65 pg/ml (197.7); post hoc *U* = 107, *P* < 0.0001, Fig. 1A). Increasing values were found in dogs in Leishvet stage IIa compared with healthy dogs and in dogs in stage IIb compared with dogs in stage IIa, but the difference was not statistically significant (post hoc *U* = 70, *P* = 0.16; post hoc *U* = 107, *df* = 4, *P* = 0.1, respectively, Fig. 1A).

Higher uMCP-1/Cr was observed in dogs in LeishVet stage IIb (median values and IQR: 9.92 × 10^−7^ (10.86 × 10^−7^), Fig. 1B), III (median values and IQR: 13.03 × 10^−7^ (9.33 × 10^−7^), Fig. 1B), and IV (median values and IQR: 24.19 × 10^−7^ (17.99 × 10^−7^), Fig. 1B) compared with healthy dogs (median values and interquartile range (IQR): 1.21 × 10^−7^ (2.08 × 10^−7^); *H* = 34.91, *df* = 4, *P* = 0.027, *P* < 0.0001, *P* < 0.0001, respectively, Fig. 1B). Furthermore, dogs in stages III and IV of LeishVet (median values and IQR: 13.03 × 10^−7^ (9.33 × 10^−7^) and 24.19 × 10^−7^ (17.99 × 10^−7^), respectively, Fig. 1B) had significantly higher uMCP-1/Cr compared with dogs in stage IIa (median values and IQR: 2.86 × 10^−7^ (2.04 × 10^−7^); post hoc *U* = 110, *P* = 0.003; post hoc *U* = 108, *P* < 0.0001, respectively, Fig. 1B). Increasing values were found in dogs in LeishVet stage IIa compared with healthy dogs and in dogs in stage IIb, but the difference was not statistically significant (post hoc *U* = 62, *P* = 0.18; post hoc *U* = 63, *P* = 0.28, respectively, Fig. 1B).

**Fig. 2:**
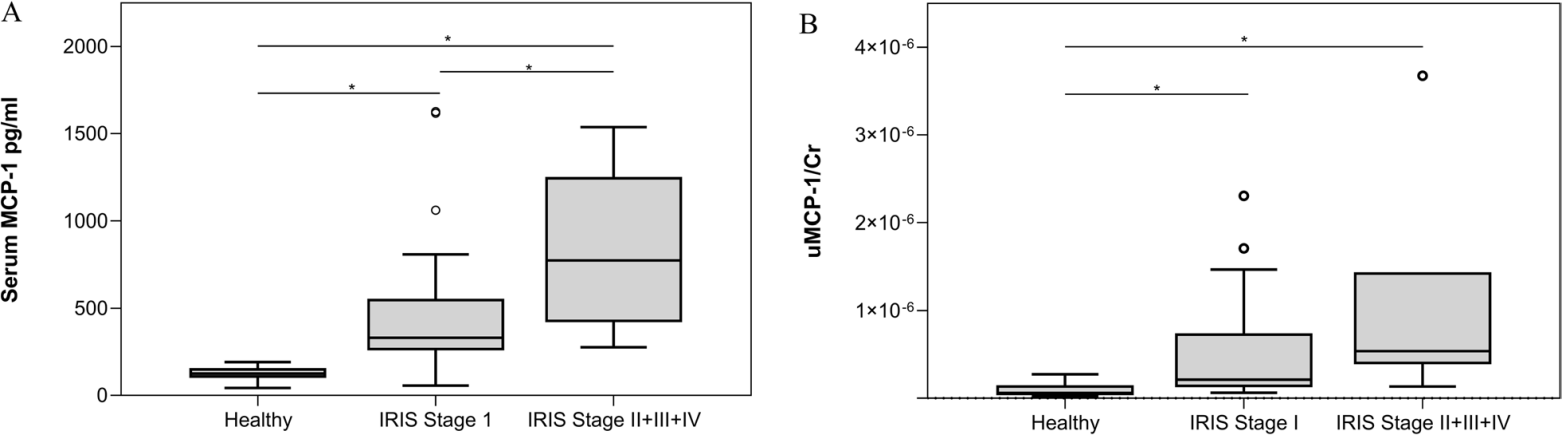
Box plots showing **A** serum MCP-1 and **B** uMCP-1/Cr in healthy dogs and dogs with leishmaniosis according to the IRIS staging to which they belong. *Statistically significant difference between the median of each group.

According to IRIS stage, a higher serum MCP-1 was observed in dogs in stage I (median values and IQR: 331 pg/ml (247.27), Fig. 2A), and in dogs in stages II + III + IV (median values and IQR: 775.3 pg/ml (748.01), Fig. 2A) compared with healthy dogs (median values and IQR: 124.83 pg/ml (48.05); *H* = 28.55, *df* = 2, *P* < 0.0001, Fig. 2A). Furthermore, serum MCP-1/Cr was significantly higher in dogs in stages II + III + IV compared with dogs in stage I (*H* = 28.55, *df* = 2, *P* < 0.0001, Fig. 2A).

Dogs with higher uMCP-1/Cr were detected in stage I and in stages II + III + IV of IRIS (median values and IQR: 4.27 × 10^−7^ (17.99 × 10^−7^) and 10.75 × 10^−7^ (16.31 × 10^−7^), respectively, Fig. 2B) compared to healthy dogs (median and IQR: 1.21 × 10^−7^ (11.59 × 10^−7^);* H* = 22.51, *df* = 2, *P* < 0.0001, respectively, Fig. 2B). Despite the higher values of uMCP-1/Cr in dogs’ stages II + III + IV of IRIS compared with dogs in stage I, the difference was not statistically significant (*H* = 22.51, *df* = 2, *P* = 0.18, Fig. 2B).

**Fig. 3:**
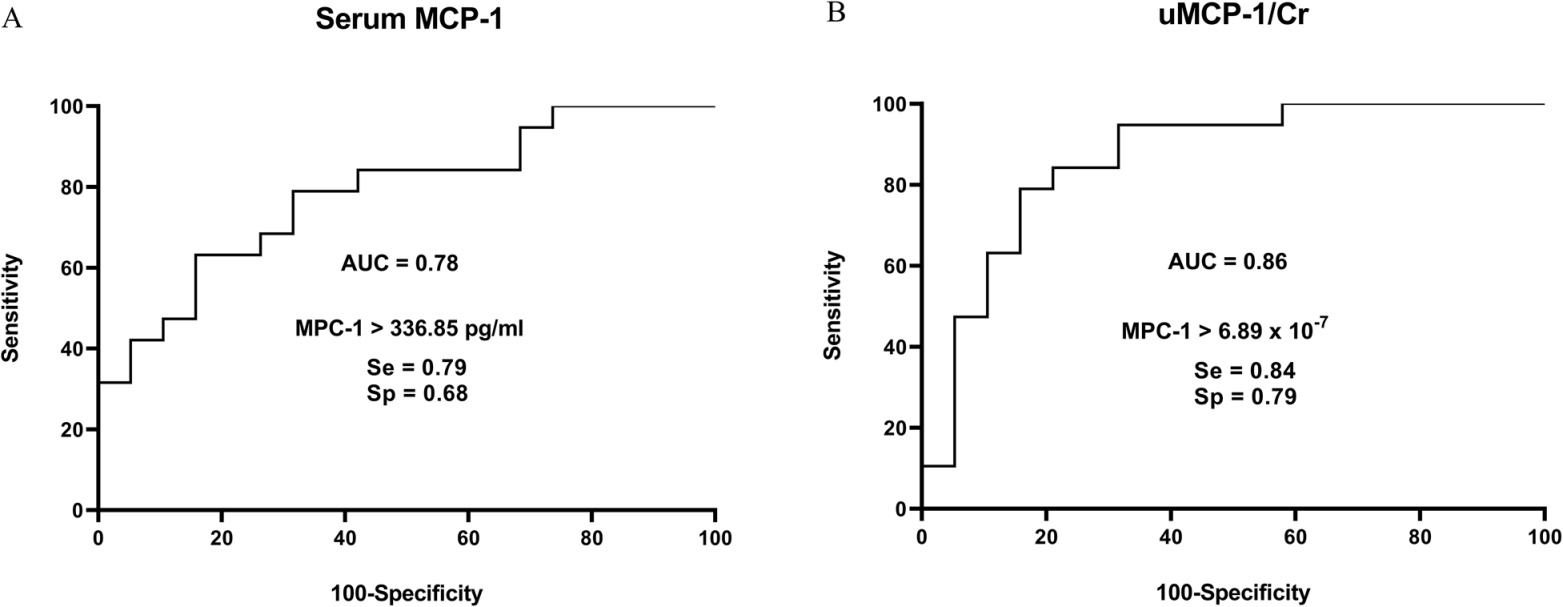
Receiver operating characteristic curve and area under the curve (AUC) to differentiate between dogs with leishmaniosis with moderate disease (LeishVet IIa + IIb) and with severe to very severe disease (LeishVet III + IV) using **A** serum MCP-1 and **B** uMCP-1/Cr. Younden index cut-off with their associated sensitivity (Se) and specificity (Sp) is presented for each curve

ROC curves were calculated to differentiate between dogs with leishmaniosis with moderate disease (group 1) from those with severe to very severe disease (group 2) at the time of diagnosis. Serum MCP-1 showed a fair accuracy (AUC = 0.78, 95% CI 0.64–0.93; *P* = 0.002, Fig. 3A) and the threshold value was 336.85 with a sensitivity of 79% and a specificity of 68%. uMCP-1/Cr showed a good accuracy (AUC = 0.86, 95% CI 0.74–0.99; *P* < 0.0001, Fig. 3B), and the threshold value was 6.9 × 10^−7^ with a sensitivity of 84% and a specificity of 79%. Of all variables studied, uAm/Cr was the best parameter to detect the severity of the disease with statistical significance at the time of diagnosis (Table 8). On the basis of the ROC curves, uMCP-1/Cr, TIBC, and Alb were shown to be good markers to discriminate between group 1 and group 2 (Table 4; Fig. 3B).

**Table 4:** Area under the curve of various variables between group 1 and group 2

Variables	AUC (95% CI)	*P*-value	k	Se, Sp
uAm/Cr	0.92	< 0.0001	300.7	79%, 100%
	(0.83–1)			
TIBC	0.86	< 0.0001	307.5	84%, 79%
	(0.73–0.98)			
Alb	0.85	< 0.0001	2.15	58%, 100%
	(0.72–0.98)			
CRP	0.80	0.015	2.1	68%, 79%
	(0.62–0.97)			
SDMA	0.80	0.002	15	79%, 68%
	(0.58–0.93)			
Iron	0.76	0.007	76.5	68%, 89%
	(0.59–0.92)			
USG	0.65	0.108	1034	74%, 68%
	(0.47–0.84)			
Glob	0.64	0.15	5.65	52%, 79%
	(0.46–0.82)			
PON-1	0.60	0.293	3.11	58%, 68%
	(0.41–0.79)			
Ft	0.56	0.549	294	94%, 26%
	(0.37–0.75)			
Hp	0.53	0.770	53	89%, 26%
	(0.34–0.72)			

uAm/Cr, urinary amylase to creatinine ratio; TIBC, total iron binding capacity; Alb, albumin; CRP, C-reactive protein; SDMA, symmetric-dimethylarginine; USG, urine specific gravity; Glob, globulins; PON-1, paraoxonase-1; Ft, ferritin, Hp, haptoglobin; AUC, area under the curve; CI, confidence interval; k, threshold value; Se, sensitivity; Sp, specificity

In healthy dogs, there was a weak positive correlation between serum MCP-1 and uMCP-1/Cr (Pearson’s coefficient correlation, *r* = 0.22, *P* < 0.001), while in sick dogs with leishmaniosis, a moderate positive correlation between these two markers was found (*r* = 0.42, *P* < 0.0001).

There was no significant correlation between serum MCP-1 and Alb in healthy dogs (*r* = 0.19, *P* = 0.43) and a moderate negative correlation in sick dogs (*r* = −0.43; *P* = 0.007). When considering the association between serum MCP-1 and Glob, a positive correlation was found in healthy dogs (*r* = 0.52, *P* = 0.02) and no correlation was found in dogs with leishmaniosis (*r* = 0.16, *P* = 0.35, respectively).

In healthy dogs, no significant correlation was found between serum MCP-1 and various and renal markers except urea (*r* = 0.54, *P* < 0.0005), while a significant positive correlation between serum MCP-1 and inflammatory markers, such as iron and TIBC, was found (*r* = 0.47, *P* < 0.005; *r* = 0.61, *P* < 0.0005, respectively, Table 5). In dogs with leishmaniosis there was a moderate positive correlation between serum MCP-1 and CRP, urea, Cr, and SDMA (*r* = 0.49, *P* < 0.0005; *r* = 0.50, *P* < 0.0001; *r* = 0.47, *P* < 0.005; *r* = 0.49, *P* < 0.0005, respectively) and a negative correlation with TIBC (*r* = −0.42, *P* < 0.0005, Table 6).

**Table 5:** Correlation between serum MCP-1 and various inflammatory and renal markers in healthy dogs

	sMCP-1	CRP	Ft	PON-1	Iron	TIBC	Urea	Cr	SDMA
sMCP-1		0.09***	−0.12*	0.05	0.47**	0.61***	0.54***	−0.03**	0.18***
CRP	0.09***		0.12*	0.01	−0.38***	−0.06**	0.14*	0.08	−0.05**
Ft	−0.12*	0.12*		−0.14*	−0.13	−0.1	−0.26	0.17	−0.11
PON-1	0.05	0.01	−0.14*		−0.38*	−0.06	0.05	−0.45	0.43
Iron	0.47**	−0.38***	−0.13**	0.38*		0.46***	0.21*	−0.06	0.26**
TIBC	0.61***	−0.06**	−0.1	−0.06	0.46***		0.44***	0.02**	0.10***
Urea	0.54***	0.14*	−0.26	0.05	0.21*	0.44***		0.44***	0.42**
Cr	−0.03**	0.08	0.17	−0.45	−0.06	0.02**	0.44***		0.29***
SDMA	0.18***	−0.05*	−0.11	0.43	0.26**	0.10***	0.42***	0.29***	

Pearson test (*r*); bold: data considered significant with *P* < 0.05 and *r* ≥ 0.4

sMCP-1, serum monocytes chemoattractant protein-1; CRP, C-reactive protein; Ft, ferritin; PON-1, paraoxonase-1; TIBC, total iron binding capacity; Cr, creatinine; SDMA, symmetric-dimethylarginine

^*^P < 0.05, **P < 0.005, ***P < 0.0005

**Table 6:** Correlation between serum MCP-1 and various inflammatory and renal markers in leishmaniotic dogs

	sMCP-1	CRP	Ft	PON-1	Iron	TIBC	Urea	Cr	SDMA
sMCP-1		0.49***	0.07*	−0.24	−0.30**	−0.42***	0.50***	0.47**	0.49***
CRP	0.49***		0.13*	−0.16	−0.40**	−0.41**	0.24*	−0.39***	0.2*
Ft	0.07*	0.13*		−0.30*	−0.29**	−0.06	−0.12	−0.10	−0.11
PON-1	−0.24	−0.16	−0.30*		0.26*	0.25	0.05	−0.03	0.08
Iron	−0.30**	−0.40**	−0.29**	0.26*		0.45***	0.29*	−0.25	−0.29**
TIBC	−0.42**	−0.41**	−0.06	0.25	0.45***		−0.53***	−0.42**	−0.47***
Urea	0.50***	0.24*	−0.12	0.05	0.29*	−0.53***		0.91***	0.72***
Cr	0.47**	0.10	−0.10	−0.03	−0.25	−0.42**	0.91***		0.72***
SDMA	0.49***	0.20*	−0.11	0.08	−0.29**	−0.47***	0.70***	0.72***	

Pearson test (*r*); bold: data considered significant at *P* < 0.05 and *r* ≥ 0.4

sMCP-1, serum monocytes chemoattractant protein-1; CRP, C-reactive protein; Ft, ferritin; PON-1, paraoxonase-1; TIBC, total iron binding capacity; Cr, creatinine; SDMA, symmetric- dimethylarginine

^*^P < 0.05, **P < 0.005, ***P < 0.0005

In healthy dogs, there was a moderate negative correlation between the uMCP-1/Cr and USG (*r* = −0.45, *P* < 0.0005, Table 7). When considering dogs with leishmaniosis, a negative correlation was still present between the uMCP-1/Cr and USG (*r* = −0.49, *P* < 0.0005) and a strong positive correlation between the uMCP-1/Cr and UPC (*r* = 0.72, *P* < 0.0005), and uMCP-1/Cr and uAm/Cr was observed (*r* = 0.74, *P* < 0.0005, Table 8). Interestingly, there was also a positive correlation between the uMCP-1/Cr with SDMA but not with Cr (*r* = 0.44, *P* < 0.0005; *r* = 0.17, *P* = 0.30, respectively, Table 8). Furthermore, a strong positive correlation and a very strong positive correlation were detected between Cr and SDMA and UPC and uAm/Cr (*r* = 0.72, *P* < 0.0005, *r* = 0.91, *P* < 0.0005, respectively, Table 8).

**Table 7:** Correlation between the uMCP-1 and various urinary and renal markers in healthy dogs

	uMCP-1/Cr	UPC	USG	uAm/Cr	Urea	Cr	SDMA
uMCP-1/Cr		0.18***	−0.45***	**−**0.12***	**−**0.14**	**−**0.006	**−**0.26***
UPC	0.18***		−0.34**	−0.19***	−0.50***	−0.24**	−0.31***
USG	−0.45***	−0.34**		0.19*	0.06*	−0.18*	0.06***
uAm/Cr	−0.12***	−0.19***	0.19*		−0.05***	0.10*	−0.26***
Urea	−0.14**	−0.50***	0.06*	-0.05***		0.44***	0.42**
Cr	−0.006	−0.24**	−0.18*	0.10*	0.44***		0.29***
SDMA	−0.26***	−0.31***	0.06*	−0.26*	0.42***	0.29***	

Pearson test (*r*); bold: data considered significant at *P* < 0.05 and *r* ≥ 0.4

uMCP-1/Cr, urinary MCP-1 to creatinine ratio; UPC, urine protein to creatinine ratio; USG, urine specific gravity; uAm/Cr, urinary amylase to creatinine ratio; Cr, creatinine; SDMA, symmetric- dimethylarginine

^*^P < 0.05, **P < 0.005, ***P < 0.0005

**Table 8:** Correlation between the uMCP-1 and various urinary and renal markers in leishmaniotic dogs

	uMCP-1/Cr	UPC	USG	uAm/Cr	Urea	Cr	SDMA
uMCP-1/Cr		0.72***	−0.49***	0.74***	0.31**	0.17	0.44***
UPC	0.72***		−0.32**	0.91***	−0.50***	0.54***	0.58***
USG	−0.49***	−0.32**		−0.26*	0.06*	−0.31*	−0.70***
uAm/Cr	0.74***	0.91***	−0.26*		−0.05**	0.35*	0.48***
Urea	0.31**	−050**	0.06*	−0.05**		0.44***	0.42***
Cr	0.17	0.54***	−0.31*	0.35*	0.44***		0.72***
SDMA	0.44***	0.58***	−0.70***	0.48***	0.42***	0.72***	

Pearson test (*r*); bold: data considered significant at *P* < 0.05 and *r* ≥ 0.4

uMCP-1/Cr, urinary monocytes chemoattractant protein-1 to creatinine ratio; UPC, urine protein to creatinine ratio; USG, urine specific gravity; uAm/Cr, urinary amylase to creatinine ratio; Cr, creatinine; SDMA, symmetric-dimethylarginine

^*^P < 0.05, **P < 0.005, ***P < 0.0005

## Discussion

This study described, for the first time, serum MCP-1 and uMCP-1 measurements in dogs with leishmaniosis by using an ELISA and showed a significantly increased serum MCP-1 and uMCP-1/Cr in dogs with leishmaniosis compared with healthy dogs. Monocyte chemoattractant protein-1 plays an important role in the pathophysiology of *L*. *infantum* infection and various veterinary studies have investigated the expression of MCP-1 in visceral organs and skin of dogs with clinical leishmaniosis [60–64]. The result of these previous studies was an increase in MCP-1 expression in different tissues associated with disease progression in agreement with the present study, suggesting an accumulation of infiltrating macrophages attracted by MCP-1 as a response of the immune system [60, 61]. Serum MCP-1 in dogs with leishmaniosis was increased, unlike that found in a recent human study in which serum MCP-1, evaluated with ELISA, in patients with cutaneous leishmaniosis was lower compared with the healthy control group [58]. The different result between the two studies probably had multiple reasons: first, the different clinical form of the disease, dogs with leishmaniosis had mainly systemic manifestation of *L*. *infantum* infection in contrast with human patients, which showed only localized cutaneous lesions owing to *L*. *major* or *L*. *tropica* infection. Therefore, in humans, serum MCP-1 is not elevated likely due to the fact the inflammatory process is localized and not systemic while in dogs the inflammatory process is systemic and it is well known that the immune response to infection partially depends on the specific *Leishmania* spp. involved and its virulence; and second, a considerable different host dependent component among dogs and human patients.

The increase in uMCP-1/Cr in dogs with leishmaniosis compared with healthy dogs was consistent with the results of a human study in which, at diagnosis, patients with visceral leishmaniosis had a higher uMCP-1/Cr compared with the healthy control, suggesting the presence of inflammation and incipient renal damage in the kidneys [59]. The common result can be explained by the fact that in both studies there was parasite dissemination after infection. When considering dogs with leishmaniosis based on LeishVet clinical staging and IRIS staging, dogs in LeishVet stage IIb and IRIS stage I had significantly higher serum MCP-1 and uMCP-1/Cr compared with healthy dogs. These data are important because they show the presence of systemic inflammation on one side and renal inflammation and damage on the other before a change in serum Cr, which is considered one of the main biomarkers of a reduction in renal function. Oliveira and colleagues also showed an increase in uMCP-1/Cr in the absence of an increase in serum Cr in humans, suggesting that inflammation and incipient renal damage can occur before changes in Cr in human patients with visceral leishmaniosis [59].

Interestingly, according to the clinical staging of LeishVet and the staging of IRIS, serum MCP-1 was significantly higher in dogs in stage IV of LeishVet compared with stage IIa and in dogs in stage II + III + IV of IRIS compared with stage I. These results are in line with those of a study in dogs with *B*. *canis* infection in which complicated dogs (and therefore dogs with more severe disease) had higher serum MCP-1 compared with uncomplicated dogs [30]. The dogs in LeishVet stage III (dogs with severe disease) had similar serum MCP-1 compared with dogs in LeishVet stage IIa and IIb (dogs with moderate disease), even if there was a decreasing trend in dogs in Leishvet stage IIa compared with dogs in LeishVet stage III and, an increasing trend in dogs in LeishVet stage IIb compared with dogs in LeishVet stage III. This result was surprising because a higher level of serum MCP-1 would be expected in dogs with more severe disease, but the difference in the sum of ranks was not large enough to be statistically significant at the alpha equals 0.05 level.

uMCP-1/Cr was significantly higher in dogs in stages III and IV of LeishVet compared with stage IIa. These results can be explained by the fact that MCP-1 is produced by intrinsic renal cells, mesangial, and endothelial cells, when stimulated by inflammatory inducers, such as immune complexes [75], which are more commonly encountered in dogs with leishmaniosis with more advanced stages of renal disease [65]. A recent study, evaluating collagen deposition in the kidneys caused by chronic renal inflammation secondary to leishmaniosis, showed that in dogs with leishmaniosis collagen deposition is linked to different cytokines / chemokines differentially expressed in renal tissue; among the various chemokines studied, the authors showed that MCP-1 expression levels were higher in clinically affected dogs compared with subclinical infected dogs [64]. These data seem to support the results of the present study, although it should be considered that Verçosa et al. evaluated the expression of MCP-1 in the kidneys but did not measure MCP-1 in urine [64].

Serum MCP-1 and uMCP-1/Cr were also evaluated as possible markers to discriminate the severity of the disease according to the clinical staging of LeishVet. On the basis of the analysis of the ROC curve, uMCP-1/Cr and serum MCP-1 showed a good and a fair accuracy in differentiating dogs with moderate disease (stages IIa and IIb) from dogs with severe to very severe disease (stages III and IV). These results confirmed the critical role of MCP-1 in the pathogenesis of kidney disease and the progression of kidney disease and that, especially uMCP-1/Cr, can help diagnose the severity of the disease. Among the various biomarkers studied, uAm/Cr showed excellent accuracy in discriminating between dogs with moderate disease and dogs with severe to very severe disease and, therefore it was the best marker to show the severity of leishmaniosis. Urinary MCP-1/Cr, TIBC, and Alb were also useful markers to differentiate dogs with moderate disease from dogs with severe to very severe disease. These results underlined how urinary markers, first and foremost inflammatory markers, played a fundamental role in showing the severity of leishmaniosis in dogs before changes in classic biomarkers of renal function, such as Cr. Another important aspect to consider is that although leishmaniosis is primarily a glomerular disease in dogs [76, 77], tubulointerstitial damage also occurs during the course of the disease, and uMCP-1/Cr could be used as an early biomarker of tubulointerstitial and glomerular damage as previously described [78].

In the present study, a correlation analysis was performed to evaluate whether there was an association between serum MCP-1 and various inflammatory and renal parameters. A positive correlation was found between serum MCP-1 and CRP and a negative correlation between serum MCP-1, Alb, and TIBC, respectively. The positive correlation between serum MCP-1 and CRP has already been described in dogs in various inflammatory diseases, such as inflammatory bowel disease, immune-mediated hemolytic anemia, thrombocytopenia purpura, eosinophilic pneumonia, immune-mediated arthritis, glomerular nephritis, pancreatitis, and panniculitis, in a previous study [27]. Generally, CRP may increase in response to mobilized inflammatory cytokines in acute disorder, while monocytes would mobilize classically in chronic disorder [27]. If the inflammatory stimulus persists over time, the CRP may remain elevated suggesting the presence of an active inflammation [9, 10]. On the basis of the results of the present study, an increase in CRP and serum MCP-1 could be expected to begin from the moderate stages of CanL and, therefore, during the progression of the disease. The negative correlation between serum MCP-1, Alb, and TIBC suggests the potential role of serum MCP-1 as a marker of inflammation during leishmaniosis. In a previous study, a decrease in TIBC with an increase in CRP has been identified as inflammatory markers in dogs with leishmaniosis [79]. Interestingly, in the present study, there was a negative association between TIBC and CRP in sick dogs, but the lack of a strong correlation suggests that these two markers are partially regulated independently, as suggested by Silvestrini et al. [79]. In the current study, there was also a moderate positive correlation between serum MCP-1 and urea, Cr, and SDMA suggesting that serum MCP-1 could be a potential marker of renal disease in dogs with leishmaniosis. To date, the role of serum MCP-1 as renal marker remains to be defined, as shown by the review by Liu et al. in which only few studies evaluated plasma or serum MCP-1 in human kidney diseases as a marker of risk of acute kidney injury or progression of kidney disease [80].

Among the renal function markers evaluated, there was a positive correlation between uMCP-1/Cr and SDMA, a negative correlation between uMCP-1/Cr and USG but, no correlation between uMCP-1/Cr with serum Cr. When considering markers of tubular and/or glomerular damage there was a strong positive correlation between uMCP-1/Cr and UPC and between uMCP-1/Cr and uAm/Cr. These results are very different from those of a human study on visceral leishmaniosis in which they evaluated the correlation between uMCP-1/Cr and Cr, glomerular filtration rate, UPC, and urinary albumin to creatinine ratio and only found a significant association with urinary albumin to creatinine ratio as an early biomarker of glomerular damage [78].

The general results of this study show the clinical utility of serum MCP-1 and uMCP-1/Cr in CanL to detect systemic inflammation and renal damage with the first marker and, renal inflammation and damage with the second, even if uMCP-1/Cr appears to be a better marker to identify renal damage compared with serum MCP-1. There is a need for further investigation to confirm these preliminary observations, to evaluate MCP-1 in serum and urine of healthy seropositive dogs and sick infected dogs with mild disease (LeishVet stage I) as potential markers to identify inflammation and early renal damage in the absence of established renal disease. In the future, it would also be interesting to evaluate serum MCP-1 and uMCP-1/Cr as markers for monitoring response to specific antileishmanial treatment and as prognostic markers in long-term monitoring.

This study has several limitations. The limited number of dogs studied could have reduced statistical power to detect significant differences in the variables studied (especially when the dogs were further divided into different groups according to the LeishVet and IRIS stagings). No kidney biopsy was performed in healthy dogs and dogs with leishmaniosis; therefore, it is not known in which dog there was renal disease and eventually the severity based on specific histopathological changes in the kidneys.

## Conclusions

Serum MCP-1 and uMCP-1/Cr were higher in dogs with leishmaniosis compared with healthy dogs. Furthermore, serum MCP-1 and uMCP-1/Cr were more elevated in advanced stages of the disease compared with moderate stages and, therefore can be markers of the severity of the disease process.

### Supplementary Information

Additional file 1

## Data Availability

All data supporting the main conclusions are available in the manuscript and its associated files.

## Abbreviations

Alb: Albumin
AUC: Area under the curve
CanL: Canine leishmaniosis
CBC: Complete blood count
Cr: Creatinine
CRP: C-reactive protein
ELISA: Enzyme-linked immunosorbent assay
Ft: Ferritin
Glob: Globulins
Hp: Haptoglobin
IQR: Interquartile range
IRIS: International Renal Interest Society
*L*. *infantum*: *Leishmania infantum*
MCP-1: Monocytes chemoattractant protein-1
PON-1: Paraoxonase-1
q-PCR: Real-time polymerase-chain-reaction
ROC: Receiver-operating characteristic
SBP: Systolic blood pressure
SDMA: Symmetric-dimethylarginine
TIBC: Total iron binding capacity
uAm/Cr: Urinary amylase to creatinine ratio
uCr: Urinary creatinine
uMCP-1: Urinary monocytes chemoattractant protein-1
uMCP-1/Cr: Urinary monocytes chemoattractant protein-1 to creatinine ratio
UPC: Urine protein to creatinine ratio
USG: Urine specific gravity
